# Expansion of the mutation spectrum and phenotype of *USP7*-related neurodevelopmental disorder

**DOI:** 10.3389/fnmol.2022.970649

**Published:** 2022-11-16

**Authors:** Hong Zheng, Shiyue Mei, Fuwei Li, Liwan Wei, Yanchu Wang, Jinrong Huang, Feng Zhang, Jia Huang, Yanping Liu, Weiyue Gu, Hongyan Liu

**Affiliations:** ^1^The First Affiliated Hospital of Henan University of Chinese Medicine, Zhengzhou, China; ^2^School of Pediatrics, Henan University of Chinese Medicine, Zhengzhou, China; ^3^Henan Provincial Key Laboratory of Children’s Genetics and Metabolic Diseases, Children’s Hospital Affiliated to Zhengzhou University, Zhengzhou, China; ^4^Beijing Chigene Translational Medical Research Center Co., Ltd., Beijing, China; ^5^Rugby School, Warwickshire, United Kingdom; ^6^Ganzhou Women and Children’s Health Care Hospital, Ganzhou, China; ^7^Department of Medical Genetics, Henan Provincial People’s Hospital, People’s Hospital of Zhengzhou University, People’s Hospital of Henan University, Zhengzhou, China; ^8^Department of Pediatrics, Henan Provincial People’s Hospital, People’s Hospital of Zhengzhou University, People’s Hospital of Henan University, Zhengzhou, China

**Keywords:** *USP7* gene, neurodevelopmental disorder, hao-fountain syndrome, whole exome sequencing, molecular spectrum

## Abstract

**Background:**

Hao-fountain syndrome (HAFOUS) is a neurodevelopmental syndrome characterized by global developmental and severe language delays, behavioral abnormalities (including autism), and mild dysmorphic impairment of intellectual development. It is a dominant genetic disease caused by *USP7* gene (*602519) mutations on chromosome 16p13.2. So far, only 15 cases with 14 deleterious variants in the *USP7* gene have been reported.

**Materials and methods:**

This study describes three unrelated patients with *USP7* variants. Besides, we identified novel *de novo* heterozygous *USP7* variants using trio-whole exome sequencing and verified by Sanger sequencing. Furthermore, clinical characteristics were evaluated by reviewing the medical records.

**Results:**

The three identified variants, i.e., one frameshift variant (c.247_250del, p.Glu83Argfs × 18) and two missense variants (c.992A > G, p.Tyr331Cys; c.835T > G, p.Leu279Val) are unreported. The predominant clinical manifestations of the three patients included: DD/ID; language impairment; abnormal behavior; abnormal brain magnetic resonance (dilation of lateral ventricles, dilation of Virchow-Robin spaces, dilated the third ventricle, abnormal cerebral white matter morphology in bilateral occipital lobes, hypodysplasia of the corpus callosum, arachnoid cyst, delayed myelination, and widened subarachnoid space); some also had facial abnormalities.

**Conclusion:**

In summary, DD/ID is the most prevalent clinical phenotype of HAFOUS, although some patients also exhibit language and behavioral abnormalities. For the first time in China, we identified three variants of the *USP7* gene using whole-genome sequence data. This work expands the *USP7* gene mutation spectrum and provides additional clinical data on the clinical phenotype of HAFOUS.

## Introduction

Hao-fountain syndrome (HAFOUS, #616863) is a neurodevelopmental syndrome characterized by impaired intellectual development, including global developmental and language delays, behavioral abnormalities, and mild deformities. Other variable features include: hypotonia, feeding problems, delayed walking with an unstable gait, hypogonadism in males, and eye abnormalities, including strabismus. Patients develop epilepsy; others present mild white matter abnormalities on brain imaging (Fountain et al., 2019).

Heterozygous variants in the *USP7* gene cause HAFOUS. Notably, the *USP7* gene is located in chromosome 16p13.2, has 31 exons, and encodes Ubiquitin carboxyl-terminal hydrolase 7 protein comprising 1,102 amino acids. The *USP7* gene encodes a deubiquitinase, a component of the *MAGEL2* (605283)/*TRIM27* (602165) ubiquitin ligase complex. Moreover, the *USP7* gene is essential for WASH (613632)-mediated endosomal actin assembly and protein recycling. Only 15 HAFOUS cases with 14 deleterious point variants in the *USP7* gene have been reported in two articles. Hao et al. (2015) identified a novel heterozygous nonsense mutation (*Tyr143Ter*) in the *USP7* gene of a 13-year-old girl diagnosed with HAFOUS (Fountain et al., 2019). Single-allele deficiency is anticipated despite the absence of direct functional studies of this variant or research in patient cells. However, *in vitro* knockdown of the *USP7* gene in HeLa cells reduced protein levels of *TRIM27* (602165), endosomal protein recycling, and F-actin accumulation (Hao et al., 2015). Fountain et al. (2019) reported 13 *de novo* heterozygous point variants in the *USP7* gene among 14 neurodevelopmental disorder patients. Out of the 13 variants, 8 were missense variants, 2 frameshifts, 2 splice sites, and 1 nonsense variant. However, no significant genotype/phenotype correlations were noted among the affected patients with *USP7* variants and variable clinical expression.

Of note, whole exome sequencing is a significant method for the precise and cost-effective detection of pathogenic variants in Mendelian disorders (Han et al., 2020; Zhang et al., 2020). Here, using whole-exome sequencing (WES), we identified three novel *USP7* pathogenic variants in three Chinese male HAFOUS patients. We collected the clinical features of these patients, summarized, and generalized their phenotypes. Besides, previously reported cases were reviewed. Our findings improve our understanding of the molecular and clinical *USP7*-associated phenotype.

## Materials and methods

Before genetic analysis and access to clinical data, written informed consent was obtained from the patient’s parents. This study was approved by the Ethics Committee of Henan Province People’s Hospital. All three patients underwent clinical evaluations by a pediatric neurologist and medical geneticist.

### Whole exome sequencing and annotation analysis

Whole exome sequencing was performed on DNA samples obtained from leukocyte specimens. Trio-WES was conducted on all patients and parents. Exome capture was carried out using the xGen Exome Research Panel v1.0 (IDT, IA, USA) (P1), xGen Exome Research Panel v2.0 (IDT, IA, USA) (P2 and P3), and target enrichment kits. Paired-end reads were utilized for sequencing on the Illumina NovaSeq6000 and BGI DNBSEQ-T7 platforms.

**FIGURE 1:**
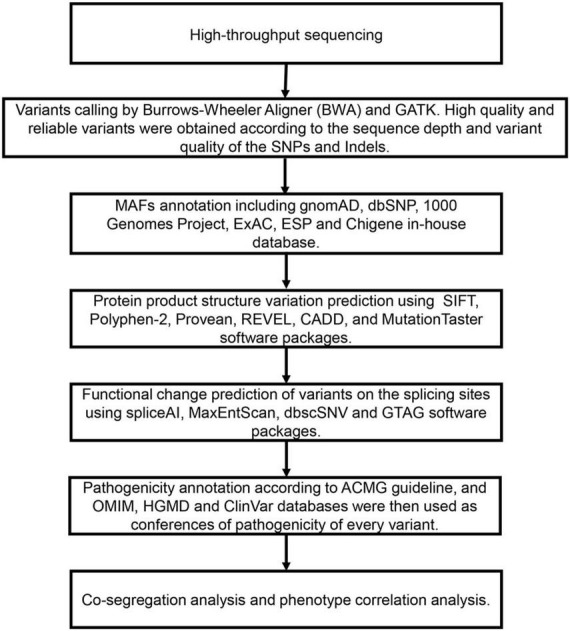
The filtering and analysis strategy and analysis process of variations screening from Trio-WES data.

Trio-WES and Variant Assessment were analyzed as follows (Figure 1).

After the removal of adapters and low-quality reads, the resultant clean data were aligned to the NCBI human reference genome (hgl9) using the Burrows-Wheeler alignment (BWA). The variants (SNPs and indels) were called using GATK. Libraries were sequenced to an average depth of × 100, and each sample had at least 95% of target bases sequenced by 20 reads. Table 1 shows the quality control of whole exome sequencing.

**TABLE 1:** Quality control data of whole exome sequencing.

ID subject	P1	P1-father	P1-mother	P2	P2-father	P2-mother	P3	P3-father	P3-mother
Human whole-exon capture probes	xGen^®^ Exome Research Panel v1.0	xGen^®^ Exome Research Panel v1.0	xGen^®^ Exome Research Panel v1.0	xGen^®^ Exome Research Panel v2.0	xGen^®^ Exome Research Panel v2.0	xGen^®^ Exome Research Panel v2.0	xGen^®^ Exome Research Panel v2.0	xGen^®^ Exome Research Panel v2.0	xGen^®^ Exome Research Panel v2.0
Sequencing platform	Illumina NovaSeq6000	Illumina NovaSeq6000	Illumina NovaSeq6000	BGI DNBSEQ-T7	BGI DNBSEQ-T7	BGI DNBSEQ-T7	BGI DNBSEQ-T7	BGI DNBSEQ-T7	BGI DNBSEQ-T7
Raw data(G)	6.35	9.74	11.18	10.11	8.29	9.45	15.29	12.06	10.6
Fraction of mapped data	99.91%	99.95%	99.95%	99.79%	99.80%	99.85%	99.75%	99.78%	99.79%
Fraction of PCR duplicate reads	31.63%	33.75%	35.73%	8.00%	8.35%	9.04%	7.70%	7.27%	7.71%
Fraction of target reads in all reads	81.41%	80.85%	79.54%	67.96%	67.51%	72.75%	64.60%	71.46%	64.75%
Average depth	93.42	142.32	160.37	115.57	94.23	115.55	166.53	144.92	115.11
≥1 × Coverage	99.47%	99.45%	99.32%	99.73%	99.61%	99.27%	99.89%	99.84%	99.64%
≥2 Coverage	99.35%	99.35%	99.19%	99.62%	99.47%	99.11%	99.84%	99.79%	99.53%
≥10 × Coverage	98.25%	98.53%	98.27%	98.99%	98.65%	98.27%	99.58%	99.47%	99.00%
≥20 Coverage	96.44%	97.59%	97.45%	98.34%	97.74%	97.47%	99.27%	99.11%	98.43%
≥30 × Coverage	93.11%	96.24%	96.48%	97.46%	96.33%	96.62%	98.90%	98.68%	97.70%
≥50 Coverage	80.15%	90.91%	92.84%	93.54%	88.55%	93.51%	97.66%	97.03%	94.46%

The databases for MAFs annotation included: gnomAD, dbSNP, 1000 Genomes Project, ExAC, ESP, and an in-house database (contains WES data from 71,405 individuals). The variants were predicted to the effects on protein product structure by SIFT, Polyphen-2, Provean, REVEL, CADD, and MutationTaster. spliceAI, MaxEntScan, dbscSNV, and GTAG software packages were used to predict a functional change of variants on the splicing sites. As a prioritized pathogenicity annotation to the American College of Medical Genetics and Genomics (ACMG) criteria and guidelines (Richards et al., 2015), OMIM, HGMD, and ClinVar databases were used as conferences of the pathogenicity of every variant. We searched the affected patients and their parents for possible causal variants that matched clinical phenotypes, such as *de novo*, recessive, compound heterozygous, or inherited X-linked variants. Sanger sequencing confirmed the *de novo* occurrence of all *USP7* gene variants from each patient. Sanger sequencing was carried out using the primers: 247-F1: GCTGATCAAATTTGGCTTCCAGTA, 247-R1: AGCACTCTGAGTTTTGGACTTCAT; 992-F2: ATGCCA TTAGATACACACAACACT, 992-R2: TGGTGGTATGTGGCT ACCAG; 835-F3: AGCCTCCCTAAATTAAGTGCCTAC, 835-R3: TCGTTAAAAGCCCTAACAACAACC. Variant annotation was based on the *USP7* gene transcript NM_003470.

### Computational structural modeling

The variant sites were mapped onto the protein structure (PBD: 2F1Z) to evaluate the effect of the identified missense variants on Ubiquitin carboxyl-terminal hydrolase 7.

## Results

### Clinical features

**FIGURE 2:**
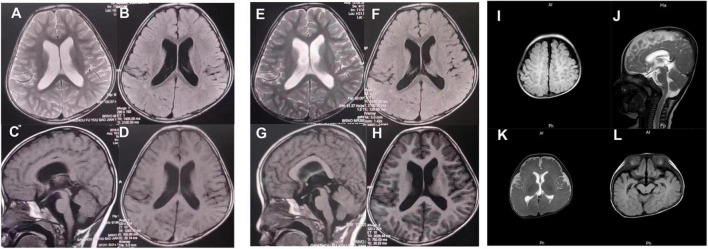
At the age of 4 years, 6 months **(A–D)**, and 5 years 1 month **(E–H)**, brain MRI (3T) of patient 1 showed lateral ventricles dilation, dilation of Virchow-Robin spaces, and dilated the third ventricle. **(I–L)** Brain MRI (3T) of patient 3 revealed delayed myelination and a widened subarachnoid space.

Patient 1 (P1) was a 5 years and 1 month old boy born to healthy, non-consanguineous parents. The patient could sit at 7 months and walk at 16, respectively. Facial features include a depressed nasal bridge, abnormal dental pulp morphology, narrow palpebral fissure, and an abnormal pinna. At 2 years, the child uttered the first words; by 4 years, he could speak only single words. The patient presented with impaired social interactions and had suboptimal self-care ability. The patient underwent surgery for hydrocele testis at the age of 2. Brain MRI at 4 years 6 months and 5 years 1 month revealed lateral ventricles dilation, dilation of Virchow-Robin spaces, and the third ventricle (Figures 2A–H).

Patient 2 (P2) was a 22-month-old boy admitted to the hospital with painful urination with no obvious cause. Laboratory test results showed increased alkaline phosphatase, lactate dehydrogenase, alpha-hydroxybutyrate dehydrogenase, lactate dehydrogenase isoenzyme 1, and decreased creatinine. Blood ammonia level was 45 μmol/L (normal range 9–30 μmol/L). Chordee and hypospadias were discovered at 18 months of age. The speech was limited to a few words at 1 year and 10 months. A brain MRI revealed abnormal cerebral white matter morphology in bilateral occipital lobes, hypodysplasia of the corpus callosum, and an arachnoid cyst.

Patient 3 (P3) was 7 months old and was born to healthy non-consanguineous parents. The patient was delivered with a weight of 2,000 g and a length of 45 cm *via* the cesarean section at the 35th week of gestation due to membrane rupture. His development delay was apparent by 4 months. At 7 months, the child could not hold his head upright or turn over. An MRI of the brain revealed delayed myelination and a widened subarachnoid space (Figures 2I–L). Echocardiography revealed a patent foramen ovale. At present, P3 is 1 year 3 months, cannot speak, can sit with support, but cannot independently stand or walk. The child has a normal height (78 cm). However, the weight is not up to the standard.

Table 2 shows the clinical features of all patients.

**TABLE 2:** The clinical phenotypes of the three patients.

Patients no.	P1	P2	P3	Our patiens	Over all cohort *USP7* variants
Gender	Male	Male	Male	3 male	9 male, 9 female
Age at evaluation	4 years 6 months	1 year 10 months	7 months		
DD/ID	+	+	+	3/3	18/18
Seizures	−	−	−	0/3	6/17
Speech delay	+	+	NA	2/2	17/17
Brain anomalies	Dilation of lateral ventricles, dilation of Virchow-Robin spaces, dilated the third ventricle,	Abnormal cerebral white matter morphology in bilateral occipital lobes, hypodysplasia of the corpus callosum, arachnoid cyst	Delayed myelination, widened subarachnoid space	3/3	11/14
Behavioral abnormalities	Impaired social interactions	–	Poor eye contact	2/3	7/16
Facial dysmorphism	Depressed nasal bridge, abnormal dental pulp morphology, narrow palpebral fissure, abnormality of the pinna	−	−	1/3	15/17
Genitourinary abnormalities	Hydrocele testis	Chordee, Hypospadias	−	2/3	4/12
Others	−	−	Patent foramen ovale		

### Molecular findings

**FIGURE 3:**
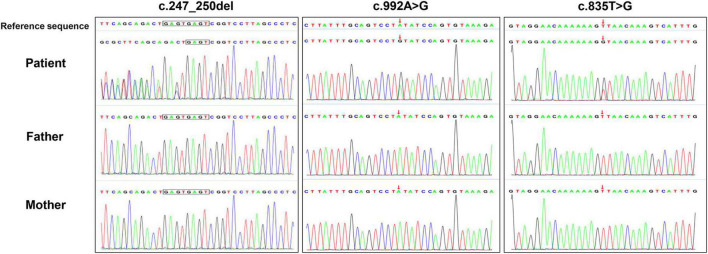
The *de novo USP7* variants were confirmed using Sanger sequencing.

**FIGURE 4:**
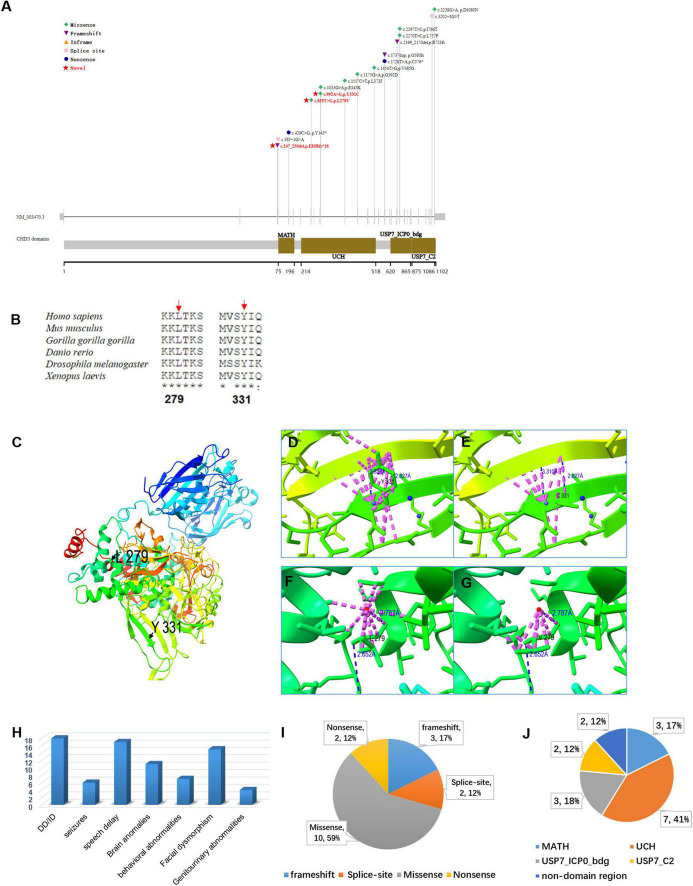
**(A)** A report on the distribution of *USP7* gene variants and protein domain pattern map in history. Variations in red font indicate loci discovered. **(B)** The conservation analysis of *USP7* amino acid sequence in multiple species on missense variants (p.Leu279Val and p.Tyr331Cys). **(C)** The panoramic 3D map of wild-type *USP7* protein. Although the p.Tyr331Cys (one hydrogen bond with Gly392) **(D,E)** and p.Leu279Val (three hydrogen bonds with Thr276, Ser282, and Phe283) **(F,G)** and wild-type and mutant hydrogen bonds did not change, interaction between the benzene ring of TYR331 and neighboring atoms, interaction between LEU279 and neighboring atoms, the amino acid side chain following mutation changed. Interactions between atoms within 4A are represented in dashed pink, and hydrogen bonds in blue dotted lines, the red and blue dots represent water molecules; **(H)** bar graph showing the distribution of the relevant clinical features among all the subjects (20) identified as having *USP7* variants. The *y*-axis shows the number of patients. **(I)** A pie chart showing the percentage distribution of *USP7* variants. **(J)** A pie chart showing the proportion of *USP7* variants in the different functional domains.

In the three families, we identified three *de novo* variants in *USP7*, i.e., one frameshift variant and two missense variants. P1 had a frameshift variant c.247_250del (p.Glu83Argfs × 18). The missense variants c.992A > G (p.Tyr331Cys) and c.835T > G (p.Leu279Val) were identified in P2 and P3, respectively. Sanger sequencing confirmed the result of trio-WES (Figure 3). All variants had never been reported before (Figure 4A). According to ACMG guidelines, these three mutations were considered pathogenic or likely. Table 3 shows the pathogenicity evidence of the variants. These variants were excluded from the SNP gnomAD, 1000 Genomes Project, ExAC, ESP, and in-house databases. The amino acid sequence analysis of multiple species showed that all the two missense variants were located in the highly conserved regions (Figure 4B). As shown in the 3D maps of the two missense variants (Figure 4C), p.Tyr331Cys (one hydrogen bond with Gly392) and p.Leu279Val (three hydrogen bonds with Thr276, Ser282, and Phe283) wild-type and mutant hydrogen bonds remained unchanged. However, a change was evident in the interaction between the benzene ring of TYR331 and neighbor atoms, the interaction between LEU279 and neighbor atoms, and the amino acid side chain after mutation (Figures 4D–G). Changes in amino acid side chains alter the normal spatial structure and stability of proteins, thereby disrupting protein function. No additional potential pathogenic CNVs with disease segregation were identified.

**TABLE 3:** The computer hazard predictions and ACMG pathogenicity rating of the three identified variants of *USP7*.

Patient	P1	P2	P3
Coordinate (GrCh37/Hg19)	chr16:9017205	chr16:9009197	chr16:9010899
cDNA change (NM_003470)	c.247_250del	c.992A > G	c.835T > G
Protein change	p.Glu83Argfs × 18	p.Tyr331Cys	p.Leu279Val
Variant type	Frameshift	Missense	Missense
SIFT	–	Damaging (0.001)	Damaging (0.0)
Polyphen2 _HDIV	–	Probably damaging 1.0)	Probably damaging (1.0)
Provean	–	Deleterious (−7.54)	Deleterious (−2.87)
Mutationtaster	–	Disease_causing (1)	Disease_causing (1)
REVEL	–	Deleterious (0.755)	Neutral (0.400)
SpliceAI	–	–	Deleterious (0.99)
CADD	–	Deleterious (27.8)	Deleterious (28.2)
Domain	MATH	UCH	UCH
Frequencies	gnomAD, 0; ESP,0; Chigene in-house database, 0	gnomAD, 0; ESP,0; Chigene in-house database, 0	gnomAD, 0; ESP,0; Chigene in-house database, 0
ACMG	P (PVS1 + PS2 + PM2)	LP (PS2 + PM2 + PP2 + PP3)	LP (PS2 + PM2 + PP2 + PP3)

## Discussion and conclusion

The present study describes three novel patients with pathogenic/likely pathogenic missense and truncating variants in the *USP7* gene. We also reviewed 15 previously described patients (Table 2 and Figure 4H; Hao et al., 2015; Fountain et al., 2019). All the patients had global developmental delays, impaired intellectual development, and delayed speech development. Out of the 16 patients, 7 had behavioral abnormalities, including autism, autistic traits, impulsivity, compulsiveness, short temper, attention deficit hyperactivity disorder (ADHD), and aggressive and manipulative behavior; 6 patients had seizures; 15 of 17 patients had facial features, including sunken eyes and a prominent nasal septum, extending below the alar of the nose; 11 of the 14 patients had abnormal brain MRI findings with reduced white matter, enlarged ventricles, non-specific white matter abnormalities, thinning of the corpus callosum, and mildly abnormal gyri. Other variable features included hypotonia, gastroesophageal reflux disease, constipation or diarrhea, sleep disturbance, asthma, short stature, scoliosis, kyphosis, and small hands and feet. We expanded and characterized the mutational spectrum of three novel individuals with heterozygous *de novo* pathogenic variants in the *USP7* gene. Regardless of the variant type, DD/ID was the significant clinical feature among all patients, with the majority of brain abnormalities (white matter abnormalities, delayed myelination, etc.). Some patients had language and behavioral abnormalities (autism, social impairment, etc.), and some had genitourinary abnormalities.

The *USP7* protein is a component of the *MAGEL2/TRIM27* ubiquitin ligase complex essential for WASH-mediated endosomal actin assembly and protein recycling (Isomura et al., 1992; Linardopoulou et al., 2007; Schaaf et al., 2013). The stability of the actin cytoskeleton is a vital cellular function that ensures smooth cellular traffic. The Wiskott-Aldrich syndrome protein and scar homolog (WASH) complex regulate the actin cytoskeleton (Machesky and Insall, 1998). Adding multiple ubiquitins to WASH unlocks its default auto-inhibited state, thus activating it (Hao et al., 2013). Previous research has shown that the *magel2-trim27* ubiquitin ligase complex is responsible for polyubiquitination. Patients with Prader-Willi (PWS) and Schaaf-Yang syndrome, neurodevelopmental disorders, intellectual disability with attributes, autism, and low muscle tone have *magel2* functional deficits (Tacer and Potts, 2017; Duan et al., 2021).

To date, 17 *USP7* gene mutations have been identified, i.e., 10 missense, 2 nonsense, 3 frameshift, and 2 splicing mutations (Figure 4I). Figure 4J illustrates the location of the mutations, i.e., 7 of these 17 mutations are in the UCH domain, 3 in the *USP7*_ICP0_bdg domain, 3 in the MATH domain, and 2 in the *USP7*_C2 domain. Ubiquitin-specific protease 7 (*USP7*) is a deubiquitinating enzyme that causes neurodevelopmental disorders (Hao et al., 2015). *USP7* protein (Pfam: Q93009) contains the following important functional domains: MATH, UCH, USP7_ICP0_bdg, and USP_C2. MATH domain is a binding domain, originally defined by functionally unrelated domains, i.e., the region of homology between the intracellular TRAF-C domain of the TRAF protein and the C-terminal regions of the extracellular meprins A and B. Although intracellular TRAFs and extracellular meprins are functionally unrelated, they share a conserved region of approximately 180 residues known as the meprin and TRAF homology (MATH) domain (Sunnerhagen et al., 2002). Meprins are mammalian tissue-specific metalloendopeptidases of the astaxanthin family that modulate normal developmental and pathological processes by hydrolyzing various proteins (Sterchi et al., 2008). Cytokines, extracellular matrix proteins, and growth factors are substrates for meprin. They comprise five domains, i.e., an N-terminal endopeptidase domain, a MAM domain, a MATH domain, an EGF-like domain, and a C-terminal transmembrane domain. Meprin A and B form membrane-bound homotetramers, whereas the homo-oligomers of meprin A are secreted. A proteolytic site adjacent to the MATH domain (found only in meprin A) releases the protein from the membrane (Marchand et al., 1995). TRAF protein was originally isolated due to its interaction with the TNF receptor (Rothe et al., 1994). They promote cell survival by activating downstream protein kinases and, ultimately, *NF-*κ*B* and *AP-1* transcription factors. TRAF proteins comprise 3 domains, i.e., the RING finger at the N-terminus, 1 to 7 TRAF zinc fingers in the middle, and the MATH domain at the C-terminus (Kim et al., 2016). Ubiquitin carboxyl-terminal hydrolase (*UCH*), also known as Deubiquitinating enzymes (*DUBs*), is a thiol protease that recognizes and hydrolyzes the peptide bond at the C-terminal glycine of ubiquitin (Jentsch et al., 1991). *UCH* thiol proteases contain an N-terminal catalytic domain and are sometimes followed by a C-terminal extension (Eletr and Wilkinson, 2014). Ubiquitin binds to proteins and regulates protein degradation *via* the proteasome and lysosome; coordinates the cellular localization of proteins; activates and inactivates proteins; regulates protein-protein interactions (Glickman and Ciechanover, 2002; Schnell and Hicke, 2003; Mukhopadhyay and Riezman, 2007). The *USP7_ICP0*_bdg domain is one of two C-terminal domains found on the much longer ubiquitin-specific proteases. It interacts with the herpesvirus 1 *trans-*acting transcriptional protein *ICP0/VMW110*. This domain is located at the C-terminal of the ubiquitin carboxyl-terminal hydrolases (USPs). However, its function remains unclear (Holowaty et al., 2003). Pathogenic variants in the *USP7* gene appear to cluster in the UCH domain. The variants p.Tyr331Cys and p.Leu279Val are also in the UCH domain and may reduce the deubiquitinating capacity.

In conclusion, we identified three novel variants in the *USP7* gene in three Chinese males using whole-exome sequencing. Our findings enrich the mutation spectrum of the *USP7* gene and improve the understanding of HAFOUS disease. Also, this work demonstrates the importance of whole-exome sequencing in the precise diagnosis of genetic diseases.

## Data availability statement

The datasets presented in this study can be found in online repositories. The names of the repository/repositories and accession number(s) can be found below: https://www.ncbi.nlm.nih.gov/, SCV002588724, SCV002588725, and SCV002588726.

## Ethics statement

The studies involving human participants were reviewed and approved by the Ethics Committee of Henan Province People’s Hospital. Written informed consent to participate in this study was provided by the participants’ legal guardian/next of kin. Written informed consent was obtained from the individual(s), and minor(s)’ legal guardian/next of kin, for the publication of any potentially identifiable images or data included in this article.

## Author contributions

HZ and SM wrote the manuscript. FL, LW, YW, and WG were responsible for genetic analysis. JRH, FZ, JH, YL, and HL provided clinical information. HL and WG critically reviewed the manuscript. All authors read and approved the final manuscript.
